# NOP14-mediated ribosome biogenesis is required for mTORC2 activation and predicts rapamycin sensitivity

**DOI:** 10.1016/j.jbc.2024.105681

**Published:** 2024-01-23

**Authors:** Xiao Yan, Bo-Hua Kuang, Shengsuo Ma, Ruihua Wang, Jinzhong Lin, Yi-Xin Zeng, Xiaoduo Xie, Lin Feng

**Affiliations:** 1 Department of Experimental Research, State Key Laboratory of Oncology in South China, Guangdong Key Laboratory of Nasopharyngeal Carcinoma Diagnosis and Therapy, Guangdong Provincial Clinical Research Center for Cancer, Sun Yat-sen University Cancer Center, Guangzhou, China; 2 School of Medicine, Shenzhen Campus of Sun Yat-sen University, Sun Yat-sen University, Shenzhen, China; 3 Cancer Center, Union Hospital, Tongji Medical College, Huazhong University of Science and Technology, Wuhan, China; 4 State Key Laboratory of Genetic Engineering, School of Life Sciences, Zhongshan Hospital, Fudan University, Shanghai, China; 5 Shanghai Institute of Infectious Disease and Biosecurity, Fudan University, Shanghai, China; 6 Zhangjiang mRNA Innovation and Translation Center, Fudan University, Shanghai, China

**Keywords:** mTORC2, ribosome, NOP14, Akt, rapalog

## Abstract

The mechanistic target of rapamycin (mTOR) forms two distinct complexes: rapamycin-sensitive mTOR complex 1 (mTORC1) and rapamycin-insensitive mTORC2. mTORC2 primarily regulates cell survival by phosphorylating Akt, though the upstream regulation of mTORC2 remains less well-defined than that of mTORC1. In this study, we show that NOP14, a 40S ribosome biogenesis factor and a target of the mTORC1-S6K axis, plays an essential role in mTORC2 signaling. Knockdown of NOP14 led to mTORC2 inactivation and Akt destabilization. Conversely, overexpression of NOP14 stimulated mTORC2-Akt activation and enhanced cell proliferation. Fractionation and coimmunoprecipitation assays demonstrated that the mTORC2 complex was recruited to the rough endoplasmic reticulum through association with endoplasmic reticulum-bound ribosomes. *In vivo*, high levels of NOP14 correlated with poor prognosis in multiple cancer types. Notably, cancer cells with NOP14 high expression exhibit increased sensitivity to mTOR inhibitors, because the feedback activation of the PI3K-PDK1-Akt axis by mTORC1 inhibition was compensated by mTORC2 inhibition partly through NOP14 downregulation. In conclusion, our findings reveal a spatial regulation of mTORC2-Akt signaling and identify ribosome biogenesis as a potential biomarker for assessing rapalog response in cancer therapy.

The mechanistic target of rapamycin (mTOR) is an evolutionarily conserved Ser/Thr protein kinase that governs various cellular processes, including protein synthesis, cell survival, and proliferation. mTOR consists of two distinct complexes: rapamycin-sensitive mTORC1 and rapamycin-insensitive mTORC2. mTORC1 is activated by various nutrition factors, such as growth factors, glucose, and amino acids. It plays a pivotal role in translational control by phosphorylating two key effectors, ribosomal S6 kinase (S6K) and eukaryotic initiation factor 4E (eIF4E)-binding protein (4EBP). S6K phosphorylates S6, a 40S ribosomal subunit, to facilitate protein synthesis. mTORC2 comprises mTOR kinase, Rictor, Sin1, mLST8, and other subunits. Although growth factors also trigger mTORC2 activation, the upstream regulation of mTORC2 remains less well-defined than that of mTORC1. mTORC2 phosphorylates and activates AGC kinases, including Akt, SGK, and cPKC (1, 2). Among these, the prosurvival kinase Akt is the most extensively studied mTORC2 substrate in cancer development (3). mTORC2 phosphorylates Akt at two regulatory sites, Ser473 in the hydrophobic motif and Thr450 within the turn motif, and these two sites undergo phosphorylation under different temporal and spatial conditions. Thr450 phosphorylation takes place during the translation of nascent Akt polypeptides at ribosomes, which promotes Akt folding and stability, and its phosphorylation is constitutive. In contrast, Akt Ser473 phosphorylation is stimulated by growth factors. Notably, constitutively targeting Akt to the plasma membrane (PM) can bypass the requirement of mTORC2 for Ser473 but not Thr450 phosphorylation (4, 5, 6, 7). Membrane binding enables Akt activation due to the phosphatidylinositol-4,5-disphosphate (and phosphatidylinositol-3,4,5-trisphosphate (PIP3)-binding nature of the pleckstrin homology domain on Akt, and lipid binding allosterically activates Akt by enabling substrate access (8, 9). In addition to mTORC2, the PI3K pathway also plays a crucial role in Akt activation. Upon stimulation by growth factor, PI3K catalyzes the conversion of phosphatidylinositol-4,5-disphosphate to PIP3, PIP3 recruits Akt to the plasma membrane, where 3-phosphoinositide-dependent protein kinase 1 (PDK1) phosphorylates Akt at Thr308 site in the activation loop. Phosphorylation Akt on Thr308 increases Akt protein kinase activity, and Akt could autophosphorylate S473 site in a PDK1-dependent manner (10). While significant progress has been made in understanding Akt activation, the precise coordination among mTORC2, the PI3K-PDK1 axis and membrane binding for achieving spatial and temporal regulation of Akt is not fully understood.

mTORC1 regulates protein synthesis by promoting ribosome biogenesis (11). Ribosomes serve as the central protein synthesis factory within the cell. In eukaryotes, ribosomes are composed of two subunits with densities of 40S and 60S. The assembly of the pre-40S and pre-60S subunits occurs in the nucleolus. Subsequently, the pre-40S and 60S subunits are exported independently to the cytoplasm, where they undergo final maturation steps to achieve translation competence (12). Mature ribosomes exist in two states: free in the cytosol and membrane-bound, which attach to the cytosolic side of the endoplasmic reticulum (ER) membrane, creating areas called the rough ER. In terms of structure and composition, free and membrane-bound ribosomes are nearly identical, with the primary distinction being the proteins they synthesize (13). It has been reported that ribosomes activate mTORC2 through direct binding (6, 14). However, the mechanistic basis for this interaction is not completely understood, and whether ER-bound and cytosolic free ribosomes contribute equally to mTORC2 activation remains unclear.

Spatial organization of the signaling molecules plays a fundamental role in determining mTOR signaling output. For instance, mTORC1 translocates to lysosomes upon amino acid stimulation (15). A substantial body of evidence suggests that the mTORC2 complex, including mTOR, Sin1, and Rictor, functions at the ER (16, 17, 18, 19). The ER constitutes the largest endomembrane system in a cell with an extensive network connecting to all other membranes. Its functions encompass protein translation, lipid, and cholesterol synthesis, all of which also take place on the cytoplasmic side of the ER membrane (20). However, the precise mechanisms underlying mTORC2 targeting to the ER and the physiological significance of this subcellular compartmentation are still not fully elucidated.

mTOR is an attractive target for drug development due to its role as a downstream effector for many frequently mutated oncogenic pathways, including PI3K/Akt and MAPK. Consequently, mTOR hyperactivation is frequently found in human cancers. For instance, genetic alterations in the PI3K/Akt/mTOR pathways are found in 23.4% of cases of nasopharyngeal carcinomas (NPCs), a type of malignant head and neck tumor prevalent in South China and Southeast Asia (21). Rapamycin, a macrocyclic triene antibiotic, serves as an allosteric inhibitor of mTORC1. Its anticancer activity was documented in the early 1980s, leading to the approval of rapamycin analogs, known as rapalogs, for cancer therapy since the late 1990s. However, the success of rapalogs has been limited to a few specific cancer types, including metastatic renal carcinoma, advanced breast carcinomas that are either estrogen receptor-positive or HER2-negative, giant cell astrocytoma, and neuroendocrine pancreatic tumors. One reason for the failure of rapalogs in the clinic is that they are unable to block mTORC2. Another reason is the existence of feedback regulation of mTORC1 on mTORC2-Akt signaling. mTORC1 phosphorylates S6K to induce insulin receptor substrate (IRS) phosphorylation and downregulation (22). Rapalogs turn off the S6K-dependent negative feedback loop that downregulates PI3K/Akt, resulting in Akt reactivation and therapy resistance. To date, efforts to identify biomarkers for rapalog response have not been successful (23, 24).

*Nop14* is a stress-responsive gene required for the maturation of 18S rRNA and assembly of the 40S ribosomal subunit characterized in yeast (25, 26). Human NOP14 has been reported to play oncogenic roles in human cancer (27, 28); however, the underlying molecular mechanism beyond ribosome biogenesis remains unclear. In this report, we show that human NOP14, a 40S ribosome biogenesis factor whose expression is upregulated in cancer cells, plays a pivotal role in mTORC2-Akt signaling by creating ribosome-coated ER as a platform for mTORC2 activation. These findings reveal the distinctions between PI3K-PDK1-mediated PM activation and mTORC2-directed ER-coupled translation and activation of Akt.

## Results

### High levels of NOP14 confer a poor prognosis in NPC

**Figure 1:**
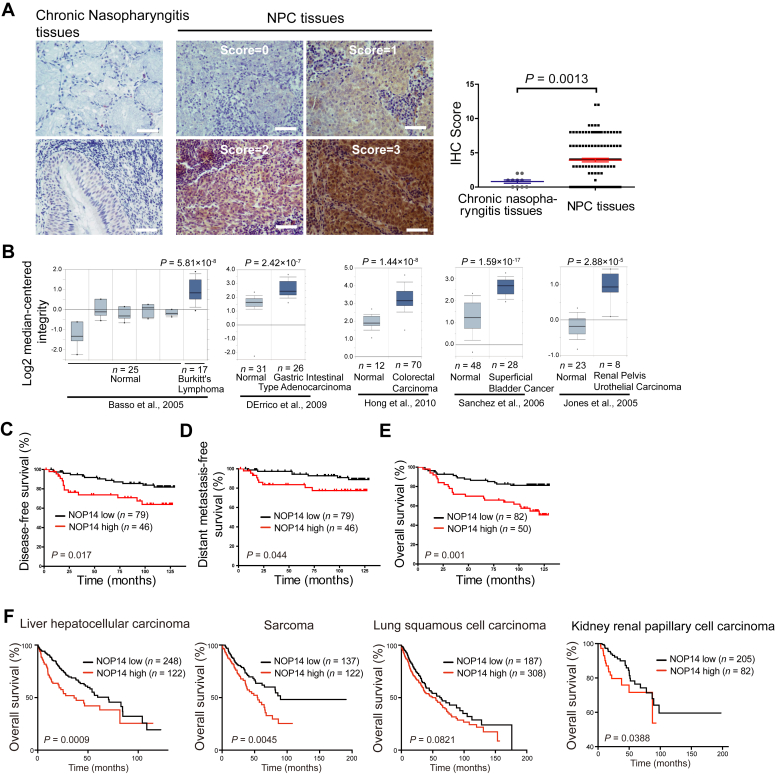
**NOP14 is overexpressed in nasopharyngeal carcinoma (NPC).***A*, (*left*). immunohistochemistry (IHC) staining of NOP14 in primary NPC lesions and chronic nasopharyngitis tissues. The scale bar represents 50 μm. (*right*) Scatterplots representing the IHC scores of NOP14 in ten chronic nasopharyngitis tissues and 132 NPC tissues. The tumor cell proportion was scored as follows: 0 (no positive tumor cells), 1 (≤30% positive tumor cells), 2 (31–50% positive tumor cells), 3 (51–75% positive tumor cells), and 4 (≥76% positive tumor cells). *B*, Oncomine box plots of NOP14 expression levels in multiple advanced human cancers. *C*, analysis of primary NPC tissues shows that patients with high NOP14 expression have poor disease-free survival (*p* = 0.017). *D*, patients with high NOP14 expression exhibited poor distant metastasis-free survival (*p* = 0.044). *E*, overall survival curves based on the analysis of 132 primary NPC tissues show that patients with high NOP14 expression have short survival times (*p* = 0.001). NOP14 low expression: score ≤4; high expression: score ≥6. *F*, Kaplan–Meier analysis of overall survival in liver hepatocellular carcinoma (370 patients), sarcoma (259 patients), lung squamous cell carcinoma (495 patients), and kidney renal papillary cell carcinoma (287 patients) stratified by NOP14 expression. The *p* values were calculated using the log-rank test. NPC, nasopharyngeal carcinoma.

Increased NOP14 expression has been reported in various human cancers (27, 28). Here, we investigated the expression of NOP14 in clinical specimens of NPC tissues. A total of 132 paraffin-embedded human NPC biopsies were subjected to immunohistochemical analysis, revealing stronger NOP14 staining in NPC lesions compared to chronic nasopharyngitis tissues (Fig. 1*A*). Moreover, Oncomine expression analysis indicated high NOP14 expression in other human cancer types (Fig. 1*B*), suggesting that elevated NOP14 expression is a characteristic feature of multiple human cancer types. The immunohistochemical staining results for NOP14 underwent statistical analysis to determine their relationship with the clinical features of NPC patients. As shown in Table S1, NOP14 expression strongly correlated with the N stage (*p* = 0.014) and clinical stage (*p* = 0.023) of the patients, while no statistically significant association was found with other factors.

Next, the correlation between NOP14 expression and patient survival (disease-free survival (DFS), distant metastasis-free survival (DMFS) and overall survival (OS)) was evaluated using Kaplan‒Meier analysis and log-rank tests. The durations of DFS, DMFS, and OS exhibited a statistically significant difference between patients with high NOP14 expression and those with low NOP14 expression, with the low NOP14 expression group showing better survival in NPC (Fig. 1, *C*–*E*) and other cancer types (Fig. 1*F*). Univariate and multivariate analyses were further conducted to assess whether NOP14 expression level serves as an independent prognostic factor for NPC patients’ outcomes. As illustrated in Table S2, NOP14 can be an independent prognostic factor for both DFS and OS; however, statistical significance was not observed for DMFS. Collectively, our data suggest that NOP14 is overexpressed in NPC and represents a novel and potentially valuable biomarker for predicting the prognosis of NPC patients.

### NOP14 promotes mTORC2-Akt signaling and is essential for cell growth

**Figure 2:**
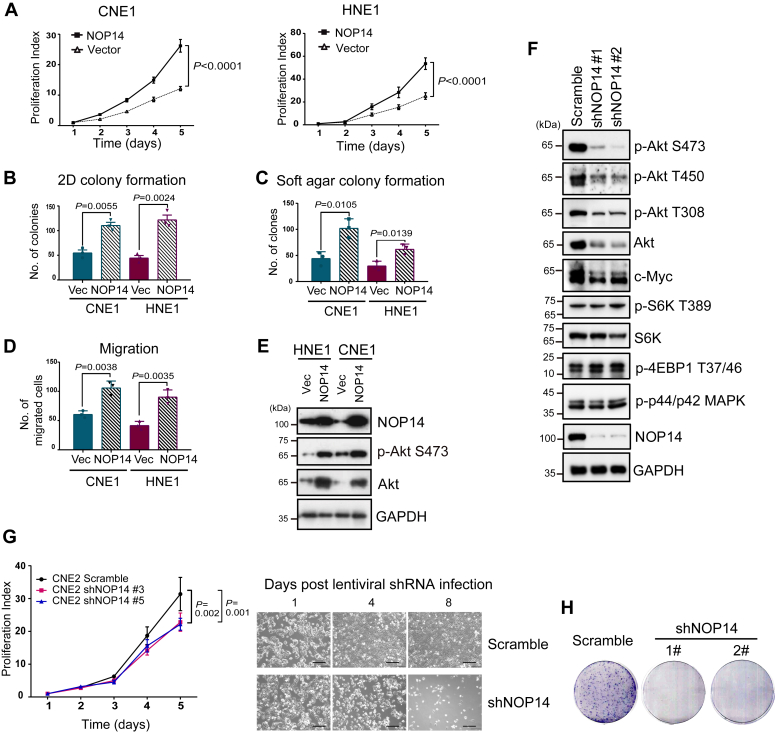
**NOP14 promotes mTORC2 signaling and cell proliferation.***A*, MTT assay showed that overexpression of NOP14 in CNE1 and HNE1 cells promoted cell proliferation. *B*, clonogenic assay showed that cells stably expressing NOP14 had higher colony forming ability. *C*, cells stably expressing NOP14 formed more colonies in soft agar than control cells. *D*, the transwell migration assay showed that NOP14 overexpression increased cell migration. *E*, total lysates of HNE1 and CNE1 control cells or stable cell lines overexpressing NOP14 were immunoblotted with the indicated antibodies. *F*, knockdown of NOP14 by two independent shRNAs inhibits mTORC2 signaling. Seventy-two hours after the second round of lentiviral transduction of shRNAs, HNE1 cells were harvested and immunoblotted with the indicated antibodies. *G*, (*top*) proliferation curve of HNE1 cells infected with scramble or NOP14-specific lentiviral shRNAs. Error bars denote standard deviation (n = 3). (*bottom*) Long-term inhibition of NOP14 caused cell death. Representative bright-field microscopy images of control and NOP14-depleted cells are shown. The scale bar represents 200 μm. *H*, cells with NOP14 depletion were unable to form colonies on plates. Data are mean ± S.D. Statistical significance was assessed using two-way ANOVA (*A* and *G*), two-sided Student’s unpaired t-tests (*B*–*D*). Individual data points are from biological replicates. mTOR, mechanistic target of rapamycin; MTT, 3-(4, 5-dimethylthiazolyl-2)-2, 5-diphenyltetrazolium bromide.

To investigate the physiological role of NOP14, we generated two cell lines, CNE1 and HNE1, that stably express NOP14. Overexpression of NOP14 in both cell lines led to a significant increase in their cell growth rates (Fig. 2*A*), colony-forming abilities in 2-D and soft agar (Fig. 2, *B* and *C*), and migration rates (Fig. 2*D*). These findings indicated that NOP14 induces cell proliferation and transformation.

We examined prosurvival pathway activities in control and NOP14 stable cells. We observed that overexpression of NOP14 markedly elevated the phosphorylation of Akt at the Ser473 site, and unexpectedly, the total levels of Akt were also elevated (Fig. 2*E*). However, the Akt mRNA level remained unaffected by NOP14 overexpression (data now shown), indicating that NOP14 exerts its control over Akt at the posttranscriptional level. To investigate the role of NOP14 in Akt under physiological conditions, we used lentivirally delivered shRNAs to deplete endogenous NOP14. Consistent with the overexpression study, downregulating NOP14 in HNE1 cells led to a reduction in the total level of Akt, as well as Akt phosphorylation at the Thr308 (A-loop), Ser473 (hydrophobic motif), and Thr450 (turn motif; TM) sites. Importantly, NOP14 knockdown had no effect on Erk phosphorylation (Phospho-p44/42 MAPK) and mTORC1-dependent S6K and 4EBP1 phosphorylation, suggesting NOP14 was not involved in mTORC1 regulation (Fig. 2*F*). As mTORC2 controls Akt stability and phosphorylation on Ser473 and Thr450 (4, 5, 6, 7), we postulated that the reduced protein and phosphorylation levels of Akt in NOP14-depleted cells might be attributed to impaired mTORC2 activity. To test this hypothesis, we determined another readout of mTORC2 activation, c-Myc level, as mTORC2 signaling acetylates FOXO to upregulate c-Myc (29). We found c-Myc expression was reduced in NOP14 knockdown cells (Fig. 2*F*), further supporting the notion that NOP14 is required for mTORC2 activation. In line with the overexpression data, knockdown of NOP14 dramatically reduced cell proliferation, and most cells died 1 one week after lentiviral shRNA infection (Fig. 2*G*); accordingly, the knockdown cells were unable to form colonies (Fig. 2*H*). Taken together, findings from both gain-of-function and loss-of-function studies suggest that NOP14 is required for mTORC2 activation and cell survival.

### The oncogenic roles of NOP14 are PI3K/Akt/mTOR pathway-dependent

**Figure 3:**
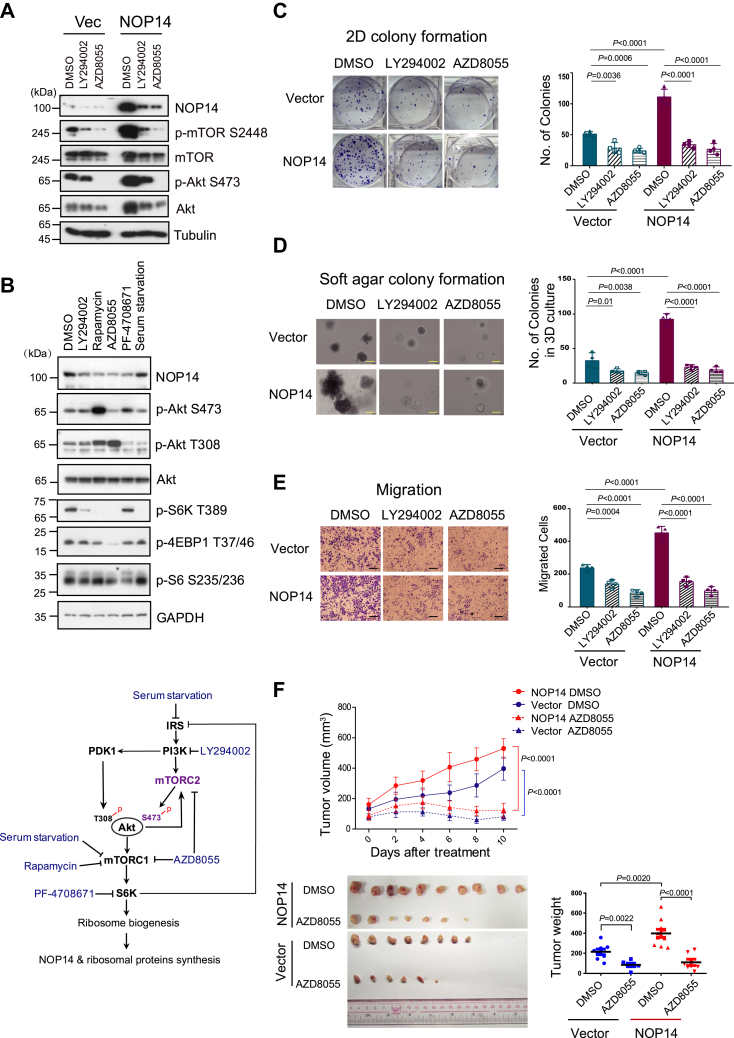
**NOP14 promotes the malignant transformation of NPC cells *via* the PI3K/Akt/mTOR pathway.***A*, control or NOP14-overexpressing CNE1 cells were treated with DMSO, 10 μM LY294002 or 0.5 μM AZD8055 for 24 h. Cell lysates were immunoblotted with the indicated antibodies. *B*, (*top*) HNE1 cells were treated with 10 μM LY294002, 1 μM rapamycin, 0.1 μM AZD8055, or 10 μM PF-4708671 or starved for 24 h. Lysates were subjected to immunoblot analysis with the indicated antibodies. (*bottom*) schematic showing the PI3K/Akt/mTOR network. *C*–*E*, inhibition of the PI3K/Akt/mTOR pathway by LY294002 (10 μM) or AZD8055 (0.1 μM) abolished NOP14 overexpression-induced growth advantages as assessed in (*C*) 2-D colony formation, (*D*) colony formation in soft agar (the scale bar represents 500 μm) and (*E*) migration assays (the scale bar represents 100 μm). (*left*) representative images of each assay; (*right*) qualification of each assay. Error bars denote standard deviation (n = 4). *F*, mice bearing CNE1 control or NOP14-overexpressing tumors were intragastrically administered DMSO or AZD8055 (15 mg/kg) every 2 days after 2 weeks of implantation. (*top*) Growth curves of the tumors after drug administration. (n = 10 mice/group). (*Bottom*, *left*) Images of tumors captured 24 days postimplantation. (*Bottom*, *right*) The tumor weight of xenografts in the different treatment groups. Data are mean ± S.D. Statistical significances were assessed using one-way ANOVA (*C*–*E*), two-way ANOVA (tumor volume in *F*) and two-sided Student’s unpaired t-tests (tumor weight in *F*). Individual data points are from biological replicates. DMSO, dimethyl sulfoxide; mTOR, mechanistic target of rapamycin; NPC, nasopharyngeal carcinomas.

A previous microarray study demonstrated that ribosomal protein S6K regulates the transcriptional activity of over 75% of ribosome biogenesis factors, including NOP14 (30). To determine whether NOP14 expression is controlled by the mTORC1-S6K pathway, control and NOP14-overexpressing cell lines were treated with the PI3K/mTOR inhibitor LY294002 or ATP-competitive mTOR inhibitor AZD8055. Strikingly, both drugs significantly reduced the protein levels of ectopically expressed NOP14, and the extent of NOP14 downregulation correlated with the reduction in Akt and mTOR phosphorylation (Fig. 3*A*). Next, the regulation of endogenous NOP14 was determined. HNE1 cells were treated with small inhibitors targeting the PI3K/Akt/mTOR pathway, including LY294002, rapamycin, AZD8055, and PF-4708671. Unlike the mTOR catalytic inhibitor AZD8055, rapamycin only inhibits mTORC1 by forming a complex with FKBP12, and PF-4708671 is an S6K inhibitor. All the above compounds, which decreased mTOR phosphorylation to varying degrees, led to a significant reduction in NOP14 protein levels. In contrast, serum starvation did not affect overall mTOR activation or NOP14 expression. Treatment with AZD8055 reduced the phosphorylation of mTOR, S6K, 4EBP1, and Akt at Ser473, while rapamycin only reduced mTOR and S6K phosphorylation but not the levels of phosphorylated 4EBP1, consistent with previous findings that rapamycin selectively inhibits the mTORC1/S6K/S6 axis but not the mTORC1/S6K/4EBP axis (31). Moreover, reactivation of Akt on S473 phosphorylation was observed following 24 h of treatment with rapamycin and PF-4708671 (Fig. 3*B*, top panel), which resulted from the elimination of mTORC1/S6K-mediated negative feedback to the IRS1-PI3K-PDK1 axis (22). The regulation of the PI3K/Akt/mTOR network by the drugs is illustrated in Figure 3*B*, bottom panel. Collectively, these findings suggest an involvement of mTORC1 signaling in the regulation of NOP14 expression.

Given that NOP14 is essential for cell survival, we further explored the role of the PI3K/Akt/mTOR pathway in NOP14-induced cell proliferation and transformation in NOP14-overexpressing cells. Indeed, inhibition of PI3K/Akt/mTOR abolished the advantages of NOP14 on cancer cell proliferation (Fig. 3*C*), colony formation in soft agar (Fig. 3*D*), cell migration (Fig. 3*E*), and tumor growth in an *in vivo* NPC xenograft model (Fig. 3*F*), consistent with the finding that NOP14 expression is mTOR dependent. Altogether, these data suggest that NOP14 plays an oncogenic role through the PI3K/Akt/mTOR pathway.

### NOP14 participates in 40S ribosome biogenesis

**Figure 4:**
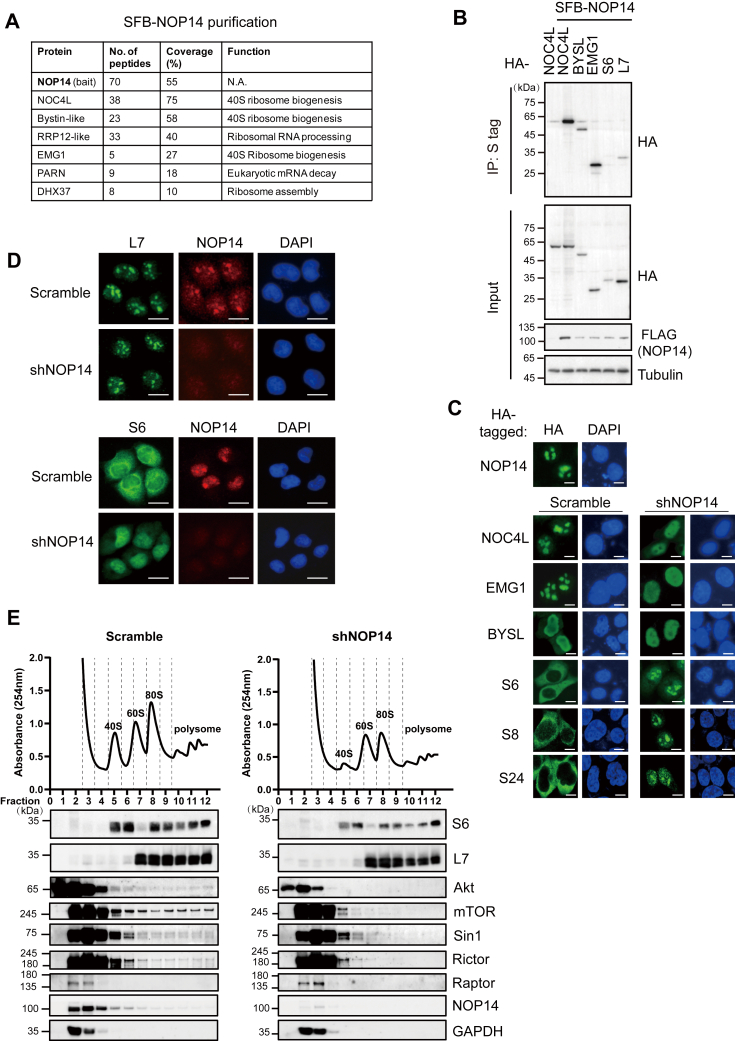
**NOP14 forms a complex with ribosome biogenesis factors to control 40S ribosome nuclear export and maturation.***A*, tandem affinity purification (TAP) of NOP14. 293T cells stably expressing SFB-tagged (S-tag, flag epitope tag, and streptavidin-binding peptide tag) NOP14 were used for TAP. Table is a summary of proteins identified by mass spectrometry analysis. *B*, coimmunoprecipitation (co-IP) of SFB-tagged NOP14 and HA-tagged prey proteins. Cell lysates were precipitated with S-protein beads and immunoblotted with the indicated antibodies. *C*, immunofluorescence images depict the subcellular localization of ectopic NOP14 and its interacting proteins with or without NOP14 knockdown. 293T cells were transfected with plasmids encoding HA-tagged constructs together with shRNA plasmids targeting the scramble sequence or NOP14 gene, and 48 h later, the cells were fixed and immunostained with anti-HA antibody. The scale bar represents 20 μm. *D*, SUNE2 cells were infected with lentiviral particles carrying shRNAs targeting the scramble sequence or NOP14 gene. Seventy-two hours postinfection, the cells were fixed and immunostained with antibodies as indicated. The scale bar represents 20 μm. *E*, 5-8F cells transfected with control or NOP14 shRNA were pretreated with cycloheximide (CHX) followed by 5 to 50% sucrose gradient sedimentation, fractions were collected and loaded onto an SDS-page gel for Western blot analysis. The *A*_254 nm_ profile (*top*) identifies the ribosome elution profiles across the sucrose gradient. HA, hemagglutinin.

To gain insights into how NOP14 operates in the mTOR pathway, we sought to reveal a comprehensive protein interaction network of NOP14. To achieve this, we performed tandem affinity purification (TAP) followed by mass spectrometry in human embryonic kidney 293T cells that stably express NOP14 tagged with S-FLAG-SBP (SFB). After two rounds of affinity purifications, NOP14-binding proteins were identified by liquid chromatography with tandem mass spectrometry analysis. The mass spectrometry data revealed that NOP14 coprecipitated with numerous small (40S) and large (60S) ribosomal subunits (Table S3). Notably, the top hits included proteins crucial for 40S ribosome biogenesis, such as nucleolar complex protein 4 homolog (NOC4L), bystin-like (BYSL), EMG1, poly(A)-specific ribonuclease (PARN), ribosomal RNA processing 12 homolog (RRP12) and DEAH-box helicase 37 (DHX37) (Fig. 4*A*). However, PI3K/Akt/mTOR network components were not found in NOP14 interactome. Previous studies in yeast has demonstrated that Noc4p (the human NOC4L ortholog) forms a stable complex with Nop14p (the human NOP14 ortholog) (26). In addition, independent high-throughput affinity purifications have uncovered interactions between human BYSL-NOP14, NOC4L, RRP12, and PARN (32), as well as BYSL-EMG1 (33) and yeast Nop14-Emg1 (25). Consistent with the results in yeast, coimmunoprecipitation experiment confirmed a strong interaction between human NOP14 and NOC4L and EMG1, a moderate interaction with BYSL, and a weak interaction with the 40S ribosomal subunit S6 and the 60S ribosomal subunit L7, indicating that NOP14 forms complexes with NOC4L-EMG1-BYSL rather than being integral ribosomes subunits (Fig. 4*B*).

Yeast *Nop14* has been reported to be essential for 40S ribosome biogenesis, similar to the functions of yeast Noc4p (26), Emg1 (25), Rrp12 (34) and human BYSL (35).We then asked whether loss of NOP14 affects the subcellular localization of its interacting proteins and ribosome biogenesis. 293T cells were transfected with hemagglutinin (HA)-tagged NOP14 and its interacting proteins along with either scrambled shRNA or shRNA targeting NOP14. Immunofluorescence data revealed that the nucleolar distribution of NOC4L and EMG1 was disrupted by NOP14 depletion, whereas BYSL was distributed in the nucleus and remained unaffected by NOP14 knockdown. Intriguingly, depletion of NOP14 resulted in strong nucleolus and nucleoplasm accumulation of ectopically expressed ribosomal small subunits, S6, S8, and S24, which were normally distributed in cytoplasm (Fig. 4*C*). We then examined the subcellular localization of endogenous 40S small ribosomal protein S6 and 60S large ribosomal protein L7. Consistent with the results of ectopic expression, endogenous S6 also underwent a translocation from the cytoplasm to the nucleus in the absence of NOP14, while the distribution of 60S ribosomal protein L7 was not affected by NOP14 knockdown (Fig. 4*D*).

To further confirm the role of NOP14 in ribosome biogenesis, we profiled cellular ribosomes by sucrose density gradients in 5-8F cells with or without NOP14 knockdown. Strikingly, cells depleted of NOP14 had a significant reduced 40S peak, as well as 80S and polysome peaks, but did not decrease the peak of free 60S subunits (Fig. 4*E*, top). Besides, in control samples, mTORC2 subunits mTOR, Sin1, and Rictor were present in fractions containing monoribosomes and polyribosomes (fractions 5–12), especially enriched in fractions with 40S ribosome subunits (fractions 5–6). In contrast, mTORC1 subunit Raptor was only detected in fractions 2 and 3, representative of free, nonribosomal lysates. Additionally, Akt showed a small but detectable presence in the ribosome containing fractions. Above observations indicated that a subset of mTORC2 and its substrate Akt were incorporated into monoribosomes and actively translating ribosomes. However, upon NOP14 depletion, the presence of mTORC2 and Akt at the ribosome-enriched fractions (fractions 5–12) were diminished (Fig. 4*E*, bottom). Thus, like its yeast ortholog, human NOP14 coordinates with other preribosomal factors to regulate the nuclear export and cytoplasmic maturation of 40S ribosomes, which is required for ribosome assembly and mTORC2-Akt association with the mature ribosomes.

### ER-bound ribosomes controlled by NOP14, but not free ribosomes, interact and activate mTORC2

**Figure 5:**
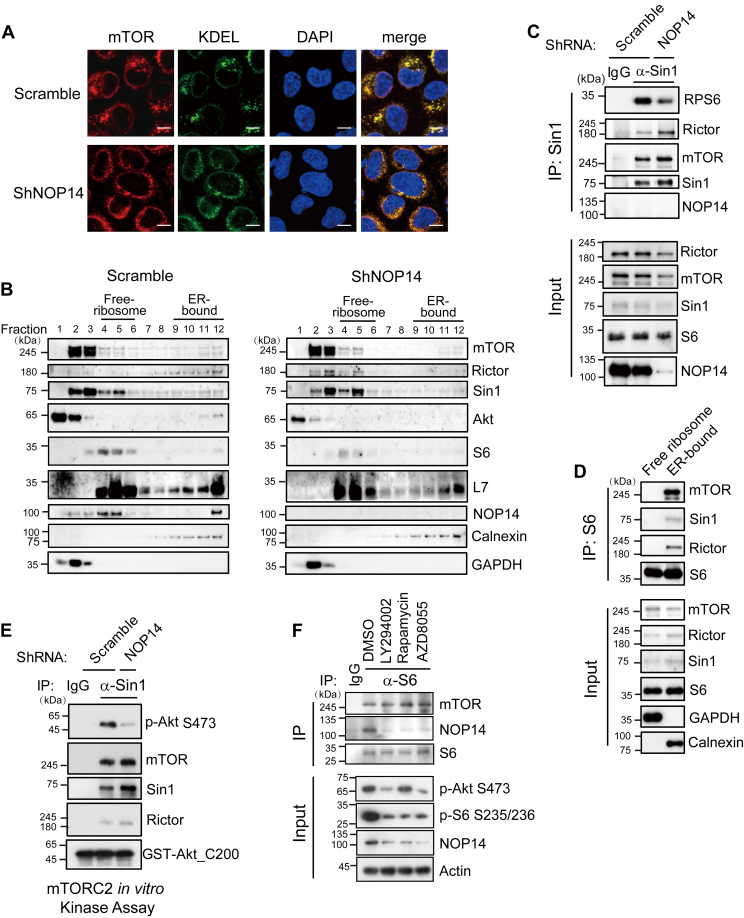
**NOP14-mediated 40S ribosome retention at the ER recruits and activates mTORC2.***A*, immunofluorescence (IF) images of mTOR kinase in control and NOP14 knockdown HNE1 cells. ER was visualized by IF staining with an anti-KDEL antibody conjugated to Alexa 488 (*green*), and mTOR kinase was stained with an anti-mTOR primary antibody followed by an Alexa 594 secondary antibody (*red*). The scale bar represents 20 μm. *B*, subcellular fractionation of ribosomes by differential centrifugation coupled with the sucrose linear gradient centrifugation. Control or NOP14 knockdown 5-8F cells were lysed by homogenizer and fractionated by velocity sedimentation. Aliquots of fractions were analyzed by immunoblotting by antibodies as indicated. *C*, mTORC2-ribosome interaction is NOP14 dependent. 293T cells were infected with control lentivirus (scrambled shRNA) or lentivirus expressing NOP14-specific shRNA. Seventy-two hours later, the cells were collected, the lysates were immunoprecipitated with anti-Sin1 antibody or control IgG in CHAPS buffer, and the coimmunoprecipitated mTORC2 components and S6 were analyzed by immunoblotting. *D*, 293T cells transfected with HA-tagged S6 were immunoprecipitated with anti-HA antibody in cytosol and organelle-enriched fractions. *E*, *in vitro* kinase assay of mTORC2 from the organelle-enriched fractions. 293T cells infected with control lentivirus or lentivirus expressing NOP14-specific shRNA were harvested and preextracted with digitonin buffer, and the pellet was then dissolved in CHAPS buffer. The supernatants were immunoprecipitated with anti-Sin1 antibody or control IgG, and the associated mTORC2 was used to phosphorylate recombinant GST-Akt1 (C-terminal 200 a.a). *F*, mTORC2-ribosome association is not disrupted by inhibitors targeting the PI3K/Akt/mTOR pathway. HNE1 cells were treated with LY294002 (10 μM), rapamycin (1 μM), or AZD8055 (0.1 μM) for 48 h, and immunoprecipitation was performed as described in (*D*). ER, endoplasmic reticulum; HA, hemagglutinin; IF, immunofluorescence; IgG, immunoglobulin G; mTOR, mechanistic target of rapamycin.

Having established the role of NOP14 in 40S ribosome biogenesis, we sought to uncover how this function is linked to mTORC2/Akt activation. Previous reports have shown that ribosomes play critical roles in mTORC2 activation through direct binding (6, 14). Ribosomes exist in two states: free in the cytosol and bound to ER. During the immunostaining of ribosomal protein S6, we observed that depletion of NOP14 not only reduced the cytoplasmic signal of S6 but also eliminated the ER-localized S6, characterized by peripheral and perinuclear dot-like staining (Fig. 4*D*). Given that several independent studies have documented the ER localization of mTOR kinase (16, 17, 18, 19) and the role of NOP14 in mTORC2 activation, we explored whether there is a correlation between ER-bound ribosomes and the ER localization of mTORC2. To test this hypothesis, subcellular localization of mTOR kinase was determined through immunofluorescence in control and NOP14-depleted cells. In agreement with previous reports, mTOR kinase colocalized with the ER marker KDEL, and the specificity of anti-mTOR antibody was confirmed by two mTOR-specific siRNAs (Fig. S1). Notably, NOP14 depletion disrupted the distribution of mTOR from perinuclear dots to the cytoplasm and perinuclear envelope, mirroring the pattern of KDEL (Fig. 5*A*).

To further elucidate the precise localization of mTORC2 and Akt, subcellular fractionation was carried out by density centrifugation. GAPDH served as a representative cytosolic protein, and calnexin is an integral ER membrane protein. Subcellular fractionation in control cells revealed the presence of mTORC2 subunits, mTOR, Rictor, and Sin1 in both free ribosomes (fractions 4–6) and the ER-enriched fractions (fractions 9–12), and Akt also existed in ER-bound fractions (fractions 11–12) besides cytosolic pool (fractions 1–3) (Fig. 5*B*, left). However, following NOP14 depletion, the distribution of Rictor, Sin1, and Akt at the ER-enriched fractions were dramatically reduced (fractions 11–12), and the reduction of the small ribosomal subunit S6 in the ER-enriched fractions were more evident than that in the free ribosome pool (fractions 11–12 *versus* fractions 4–6), suggesting the ER-bound mature 40S ribosomes could not form in the absence of NOP14. On the other hand, the distribution of 60S ribosome subunit L7 was less affected by NOP14 depletion (Fig. 5*B*, right). The results indicate that ER-bound ribosomes might be essential for mTORC2-Akt accumulation and activation at the ER.

Next, we investigated the impact of NOP14 on the integrity of the mTORC2 complex. Immunoblotting of Sin1 immunoprecipitates in control and NOP14-depleted cells suggested that the loss of NOP14 did not affect mTORC2 complex formation but diminished the association of ribosomal protein S6 with mTORC2. Notably, NOP14 was not found in the mTORC2 immunocomplex (Fig. 5*C*). As NOP14 governs ER-bound but not cytosolic free small ribosomes, we then examined which pool of ribosomes binds to mTORC2. To this end, cytoplasmic and ER-containing organelle fractions were enriched, and ribosome-mTORC2 interactions were assessed in each fraction. Surprisingly, mTOR only coimmunoprecipitated with S6 in the ER-containing organelle fraction, while such interaction was absent in the cytosol fraction, despite the presence of mTORC2 and ribosomal proteins in both fractions (Fig. 5*D*). Next, membrane-bound mTORC2 kinase activities were determined through an *in vitro* kinase assay by immunoprecipitation using anti-Sin1 antibody in membrane enriched fractions of control and NOP14-depleted cells. Recombinant Akt fragment spanning the phosphorylation sites by mTORC2 but lacking Akt kinase activity was coincubated with mTORC2 immunocomplex, and the result suggested that NOP14 knockdown reduce mTORC2 kinase activity in the *in vitro* system (Fig. 5*E*).

A previous report suggested that the mTORC2-ribosome interaction is stimulated by PI3K signaling (14), but we found that suppression of the PI3K/mTOR pathway by inhibitors did not impair mTOR-ribosome interactions, despite the decreased levels of NOP14 (Fig. 5*F*). In summary, these data suggest that membrane-bound ribosomes, but not free ribosomes, interact with and activate mTORC2, a process depends on NOP14-mediated small ribosomal subunit nuclear export and maturation on the ER.

### NOP14 levels predict sensitivity to mTOR inhibitors in NPC cells

**Figure 6:**
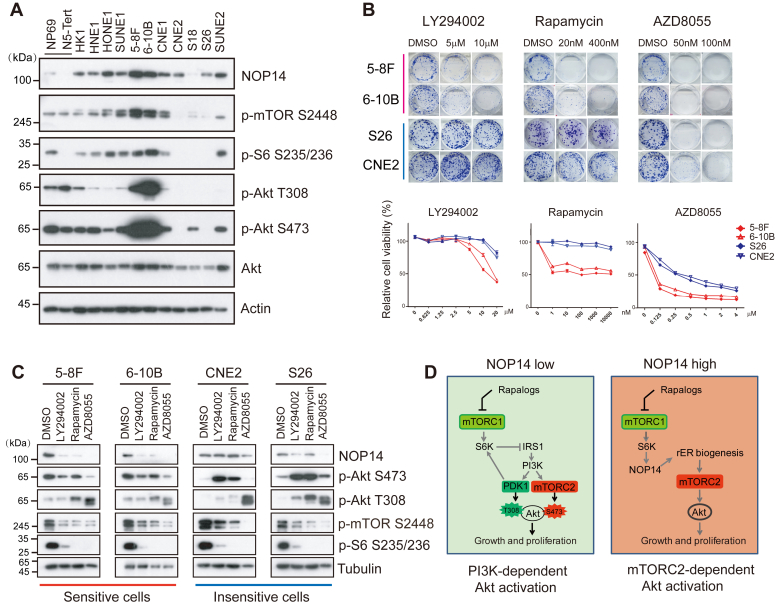
**NOP14 levels reflect sensitivity to mTOR inhibitors.***A*, NOP14 expression and PI3K/Akt/mTOR pathway activities among NPC and two normal nasopharyngeal epithelial cell lines, NP69 and N5-Tert. *B*, (*top*) Drug sensitivity of NPC cell lines in colony formation assay. (*bottom*) Drug sensitivity of NPC cell lines as determined by MTT assay for cell viability. Error bars denote standard deviation (n = 3). *C*, sensitive and insensitive NPC cells were treated with LY294002 (10 μM), rapamycin (1 μM), or AZD8055 (0.1 μM) and collected 54 h later. The lysates were immunoblotted with the indicated antibodies. *D*, a model deciphering how the NOP14 level determines the mode of Akt activation by either the PI3K pathway or the mTORC2 pathway. mTOR, mechanistic target of rapamycin; MTT, 3-(4, 5-dimethylthiazolyl-2)-2, 5-diphenyltetrazolium bromide; NPC, nasopharyngeal carcinomas.

Given the role of NOP14 in mTORC2 activation, we then sought to determine whether NOP14 levels could serve as an indicator of the responsiveness to PI3K/mTOR inhibitors in cancer cells. A range of NPC cell lines, along with two immortalized normal nasopharyngeal epithelial cell lines (NP69 and NEPC5-Tert), underwent Western blot analysis to assess NOP14 levels and mTOR pathway activity. NOP14 was observed to be upregulated in most NPC cells when compared to normal nasopharyngeal epithelial cells, with the highest levels of expression noted in 5-8F and 6-10B cancer cells. Furthermore, the expression of NOP14 displayed a positive correlation with the phosphorylation levels of mTOR, S6, and Akt (Fig. 6*A*), which was consistent with the observation that NOP14 is regulated by the mTORC1 pathway (Fig. 3).

We tried to establish the correlation between NOP14 expression and sensitivity to PI3K/Akt/mTOR inhibitors. Although inhibition of both mTORC1 and mTORC2 by AZD8055 led to cell death in all NPC cell lines we tested, the PI3K/mTOR inhibitor LY294002 and mTORC1 inhibitor rapamycin exhibited limited or no cytotoxic effects in most NPC cells, except for 5-8F and 6-10B (Fig. 6*B*). Based on the sensitivity to LY294002 and rapamycin, we classified NPC cells into two categories: sensitive (5-8F and 6–10B) and insensitive (S26 and CNE2), and evaluated their responses to PI3K/Akt/mTOR inhibitors. Prolonged treatment with AZD8055 led to feedback activation of PI3K- and PDK1-driven phosphorylation of Akt on Thr308 in both sensitive and insensitive cell lines. In contrast, inhibition of PI3K/mTOR by LY294002 or mTORC1 by rapamycin only resulted in increased phosphorylation of Akt at the Ser473 site in insensitive cells but not in sensitive cells, despite the latter possessing higher basal levels of Akt phosphorylation (Fig. 6*C*). Additionally, the reduction in NOP14 protein levels by PI3K/mTOR inhibitors was more evident in sensitive cells compared to insensitive cells (Fig. 6*C*). These data indicate that the NOP14 level plays a critical role in determining the cellular response to PI3K/mTOR inhibitors. In cells with low NOP14 expression, rapamycin and other rapalogs inhibit mTORC1-mediated S6K activation, which alleviates the negative feedback on the IRS/PI3K/PDK1 axis and leads to phosphorylation Akt on Thr308 site (36). Phosphorylation Akt on T308 increases Akt protein kinase activity, and Akt autophosphorylates Ser473 site in a PDK-1-dependent manner (10), which results in full activation of Akt and cell survival. In contrast, in cells with high levels of NOP14-mediated ribosome biogenesis, mTORC2 is highly activated by the ER-bound ribosomes, which overwhelms the PI3K/PDK1 axis in controlling Akt S473 activation. As a result, rapalogs and PI3K inhibitors induce cell death by downregulating NOP14 and the accompanying mTORC2-Akt activation. In this scenario, mTORC1 exerts positive feedback on mTORC2 (Fig. 6*D*, right). Therefore, the mode of Akt activation, whether through the plasma membrane-occurring PI3K-PDK1 pathway or through the ER-occurred mTORC2 pathway, may determine the sensitivity of cancer cells to rapalogs. Our results suggest that NOP14 could act as a valuable biomarker for predicting the response to mTOR/PI3K inhibitors.

## Discussion

mTORC2 is a pivotal complex that regulates cell proliferation and metabolism. However, in comparison to mTORC1, our understanding of mTORC2 regulation is significantly less comprehensive. In the present study, we revealed a spatial compartmentalization model of mTORC2 signaling. By studying NOP14, a nucleolar protein responsible for mediating the nuclear export and maturation of 40S ribosome at the ER, we established the role of ribosome biogenesis in mTORC2 recruitment to the surface of ER, which leads to the full activation of mTORC2, eventually promoting cell survival and uncontrolled cell growth.

It has long been noted that a majority of mTOR localizes at the ER (16, 17, 18, 19). We present evidence that the ER membrane-bound ribosomes, as opposed to the cytoplasmic pool, interacts with and activates mTORC2. The removal of ER-bound ribosomes through NOP14 knockdown leads to the detachment of mTORC2 from the ER, resulting in mTORC2 inactivation and subsequent cell death. The choice of the ER as the platform for mTORC2 activation offers several advantages. First, a locally concentrated kinase can act on substrates at much higher rates compared to diluted in the cytoplasm, and this principle holds true in numerous cases, including mTORC1 (15, 37). Second, ER binding enables the coupling of translation and cotranslational phosphorylation of Akt at the T450 site on the turn motif, which stabilizes nascent polypeptides and ensures the attainment of the proper conformation. In this way, mTORC2 effectively maintains substrate activation while exercising quality control (Fig. 6). Third, the continuous contact of the rough ER with the nucleus confers unique control over protein synthesis on the ER. The synthesis of ribosomal proteins is one of the most energy intensive processes. mTORC1 senses the nutritional requirements of cells and triggers the synthesis of ribosome biogenesis factors, including NOP14. NOP14-mediated ribosome maturation and export activates mTORC2 for the phosphorylation of substrates, such as Akt, activated Akt further stimulates the mTORC1-S6K axis, establishing a positive feedback loop between mTORC1 and mTORC2. These findings help elucidate, at least in part, the mechanism through which growth factors activate mTORC2.

The mTORC2 receptor on the ER remains unknown. Previous studies have demonstrated that the HEAT repeats of mTOR are responsible for tethering mTOR to membranes (16, 38), indicating that the HEAT repeats of mTOR might serve as a protein‒protein interaction interface for mTORC2 and ER-bound ribosomes. It is highly possible that unique components present in ER-bound ribosomes, but absent from free ribosomes, may function as mTORC2 receptors on the ER. Potential candidates include translocon proteins and other yet uncharacterized ribosome receptors on the ER (13). Further biochemical analyses or screening studies are required to pinpoint the precise mTORC2 receptor residing on the ER.

Ribosomes are abundant cellular components. It is unlikely that mTORC2 constitutively associates with ribosomes and remains activated. Indeed, only a small fraction of total ribosomes is associated with mTORC2. Previous studies have shown that PI3K signaling enhances mTORC2 activation by promoting its association with ribosomes (14). However, long-term treatment with PI3K/Akt/mTOR inhibitors did not disrupt mTORC2-ribosome interactions (Fig. 5*F*), consistent with previous observations indicating that these treatments did not alter the ER localization of the mTOR kinase (16). Factors controlling the abundance or dynamics of the mTORC2 receptor on the ER may regulate the mTORC2-ribosome association and, in turn, influence mTORC2 activation.

The function of NOP14 in ribosome biogenesis is conserved among eukaryotes. Nop14 is an essential gene in the fungi *Cryptococcus neoformans* (39), yeast species such as *Saccharomyces cerevisiae* (25), and it is also indispensable for mammalian cells. Therefore, our studies provide valuable insights into the regulatory mechanism of the TORC2 pathway in eukaryotic unicellular organisms, such as yeast. The activation of TOR in yeast has been a long-standing question, despite the TOR pathway’s being originally discovered in yeast (40). Unicellular organisms lack a mammalian-like PI3K/PTEN pathway, resulting in the absence of PIP_3_. Yeast possesses an Akt ortholog that is activated by TORC2 (41). Yeast TORC2 localizes to an as-yet-uncharacterized PM subdomain (42). It would be intriguing to investigate whether these “foci” correspond to EM-PM contact sites, which are more abundant in yeast than in mammals (20). In this regard, ER-mediated TORC2 activation may represent an ancient and highly conserved regulatory mode of TORC2. Studies on yeast ribosome biogenesis genes could provide further evidence for this model.

Rapamycin and its derivatives known as “rapalogs” have been approved for cancer therapy. Although rapamycin efficiently inhibits the mTORC1-S6K-S6 cascade, the majority of cancer cases experience relapses due to feedback activation of Akt through the PI3K-PDK1 pathway (23, 24). The intricate crosstalk and feedback regulation between the mTOR and PI3K pathways have made the search for biomarkers for rapalogs a challenging endeavor over the years. In this study, we provide a proof of concept that rapalogs are most effective in selected tumors driven by ER-activated mTORC2 signaling. In cells with elevated levels of ribosome biogenesis factors like NOP14, S6K-dependent inhibition of mTORC2 is overridden by the ER-dependent activation of mTORC2. Consequently, prolonged rapamycin treatment does not induce significant Akt reactivation and drug resistance (Fig. 6, *B* and *C*). In this setting, rapalogs not only directly inhibit mTORC1 but also indirectly suppress mTORC2 by downregulating ribosome biogenesis (Fig. 6*D*). It is of significant interest to explore whether the abundance of rough ER, marked by the abundance of NOP14 or other ribosome protein markers, can be applied to predict the sensitivity of cancer patients to rapalogs.

In head and neck cancers include NPC, the PI3K/mTOR pathway stands out as the most activated signaling pathway. Encouraging results have emerged from clinical trials using mTOR inhibitors (43). The revelation of NOP14 and its link to ER-mediated mTOR activation opens the door to assess the mode of Akt activation, a potentially valuable predictor of individual’s clinical response to rapalogs. This mechanism-driven therapeutic strategy has the potential to identify patient groups that would derive the greatest benefit from mTOR inhibition.

## Experimental procedures

### Cell culture

NP69 and NPEC5-Tert cells were maintained in keratinocyte/serum-free medium (Invitrogen), and NPC cells were maintained in Dulbecco’s Modified Eagle’s Medium (Invitrogen) with 10% fetal bovine serum (Invitrogen) at 37 °C and 5% CO2. NPC cell lines were from Prof. Mu-Sheng Zeng (Sun Yat-sen University Cancer Center). All cell lines were routinely tested and shown to be mycoplasma-free as determined by PCR-based method (16S rDNA-F: 5′-ACTCCTACGGGAGGCAGCAGTA-3′, 16S rDNA-R: 5′-TGCACCATCTGTCACTCTGTTAACCTC-3′).

### Overexpression and knockdown

To establish stable cell lines, pLVX-puro empty vector or pLVX-NOP14 was transfected into CNE1 and HNE1 cells. Puromycin (2 μg/ml)-resistant clones were selected, and the upregulation of NOP14 was verified by Western blotting.

To knockdown NOP14, shRNA targeting human NOP14 was cloned into the pLKO.1 vector. Sequences of control scramble shRNA: 5′-CCTAAGGTTAAGTCGCCCTCG-3′; two NOP14 targeting shRNA: 1#: 5′-CGGGAATGGTCTGTGTGTTAT-3′ and 2#: 5′-GCTATTTCCAACTTCCGACTT-3′. Cell transfection, packaging, and transduction of lentiviral shRNAs were performed as described previously (44), and two rounds of lentivirus infection were conducted at 24 h intervals.

### Plasmids, antibodies, and chemicals

Rictor and mTOR complementary DNA were kind gifts from Dr X. F. Steven Zheng (Rutgers Cancer Institute of New Jersey). All constructs were subcloned into pcDNA3.1 with an N-terminal HA tag. A rabbit polyclonal antibody against NOP14 (NBP2-13665) was purchased from Novus. Antibodies against phospho-Akt (Ser473) (4060), phospho-AKT (Thr450) (9267), phospho-Akt (Thr308) (9275), Akt (9272), phospho-mTOR (Ser2448) (5536), mTOR (2983), Rictor (9476), Sin1 (12860), phospho-S6K (Thr389) (9205), phospho-4EBP1 (Thr37/46) (2855); FoxO1 (2880), ribosomal protein S6 (2317), phospho-S6 (Ser235/236) (2211), ribosomal protein L7 (2415), c-Myc (13987), HA-tag (2367), S-tag (EM50105), Myc-tag (EM31105), and β-actin (4970) were purchased from Cell Signaling Technology. Anti-KDEL (Alexa Fluor 488-conjugated) (ab184819) and anti-GAPDH (ab75479) antibodies were obtained from Abcam. Anti-tubulin (sc-23948) antibody was purchased from Santa Cruz Biotechnology. A mouse monoclonal antibody against FLAG (F3165) was obtained from Sigma‒Aldrich. Peroxidase–conjugated goat anti-rabbit IgG and goat anti-mouse IgG (Amersham Pharmacia Biotech) secondary antibodies were used for Western blotting analysis.

LY294002, AZD8055, and rapamycin were all purchased from Selleck Chemicals.

### Patients and tissue samples

We analyzed 135 consecutive patients with NPC who underwent nasopharyngeal biopsy at Sun Yat-Sen University Cancer Center (Guangzhou, China) between October 2003 and November 2004. This study is reported according to the Reporting Recommendations for Tumor Marker Prognostic Studies (REMARK). Two patients were lost to follow-up, and the specimen from one patient was not available; thus, 132 patients (104 men and 28 women) were evaluated.

The age of the patients ranged from 15 to 77 years, and the mean age at the time of NPC diagnosis was 44.82 years. The pathologic stages of all NPC patients were determined based on the seventh edition of the UICC/AJCC staging system, with 7, 16, 63, and 46 having stage I, II, III, and IV tumors, respectively. The overall survival time was calculated as the date of NPC diagnosis to the time of cancer-related death or the most recent follow-up if the patient was alive. The 10-year cumulative survival rate for all patients was 68%.

Tissue samples that had been histologically and clinically confirmed were obtained from the archives of the Department of Sample Resources, Sun Yat-sen University Cancer Center; ten paraffin-embedded, noncancerous human nasopharyngeal tissues (chronic nasopharyngitis tissue), and 132 paraffin-embedded NPC tissue samples were analyzed. Consent was obtained from the patients, and the Institute Research Ethics Committee of Sun Yat-Sen University Cancer Center approved the use of the clinical samples for research purposes.

### Immunohistochemistry

Immunohistochemistry of paraffin-embedded sections was performed as previously described (24); when necessary, sections were incubated overnight at 4 °C with rabbit anti-NOP14 (1:500). The degree of immunostaining was evaluated and scored independently by two pathologists, and the staining intensity and proportion of positively stained tumor cells were used as evaluation criteria. The tumor cell proportion was scored as follows: 0 (no positive tumor cells), 1 (≤30% positive tumor cells), 2 (31–50% positive tumor cells), 3 (51–75% positive tumor cells), and 4 (≥76% positive tumor cells). Staining intensity was graded according to the following criteria: 0 (no staining), 1 (weak staining, light yellow), 2 (moderate staining, yellowish brown), and 3 (intense staining, brown). The staining index was calculated by multiplying the above two scores to yield a final score of 0, 1, 2, 3, 4, 6, 8, 9, or 12. The tumors were eventually determined as having low expression, score ≤4, or high expression, score ≥6.

### Statistical analysis

The SPSS 25 statistical software package (https://www.ibm.com/products/spss-statistics) was used for all statistical analyses. Differences among variables were evaluated by χ2 analysis or 2-tailed Student’s t tests. All data are presented as the mean ± SD, unless otherwise indicated. χ2 analysis and Fisher’s exact tests were performed to analyze correlations between NOP14 expression and clinicopathologic characteristics. Bivariate correlations between the study’s variables were evaluated using Spearman’s rank correlation coefficients.

Survival time was calculated as the date of NPC diagnosis to the time of cancer-related incidence or the most recent follow-up if the patient was alive. Survival curves were plotted using the Kaplan‒Meier method and compared using the log-rank test to determine significance. Survival data for prognostic factors were evaluated by univariate and multivariate Cox regression models. In all cases, *p* values equal to or less than 0.05 were considered to indicate a statistically significant result.

### Cell proliferation, colony formation, migration, and animal studies

Cell proliferation, colony formation in 2-D and soft agar and transwell migration assays were performed as described previously (45). For animal studies, tumor cells (5 × 10^5^ in 0.2 ml PBS) were injected subcutaneously into the left dorsal flank of 6-week-old nu/nu athymic female mice (Hunan Slac Jingda Laboratory Animal Co, Ltd). After 2 weeks of inoculation, mice were intragastrically administered vehicle or AZD8055 (15 mg/kg) every 2 days, and the tumor sizes were measured simultaneously. Mice were killed 4 weeks postinjection *via* cervical dislocation, and tumors from the two groups were extracted and weighed.

All the animal experiments were performed with the approval of Institutional Animal Care and Use Committee of Sun Yat-sen University (reference no. GZR2016-105) and the animals were handled in accordance with institutional guidelines.

Immunofluorescence microscopy was performed as described previously (37).

### Sucrose gradient-based ribosome profiling

Ribosome fractionation using sucrose density gradient centrifugation was performed as described previously with minor changes (46). Cells were pretreated with 100 μg/ml cycloheximide (CHX) for 3 min at 37 °C to stabilize ribosomes on mRNAs followed by washing using ice-cold PBS containing 100 μg/ml CHX. Cells were then lysed on ice by scraping extensively in lysis buffer (20 mM Hepes pH 7.4, 150 mM KCl, 15 mM MgCl_2_, 2% NP-40, 1× protease inhibitor cocktail (EDTA-free),10 mM RNase inhibitor, 100 μg/ml CHX, 1 mM DTT). A total of 10 *A*_254 nm_ of extract were loaded onto 5 to 50% sucrose gradient in lysis buffer followed by centrifugation at 39,000 rpm in a Beckman SW41 rotor for 3 h at 4 °C. The gradients were analyzed with a continuous gradient collector with the UV detector set to 254 nm.

### Subcellular fractionation

Free cytosolic polysomes and membrane-bound polysomes were fractionated by density centrifugation as described previously (47). Briefly, cells were homogenized in cold sucrose buffer (0.25 M sucrose, 10 mM Tris–HCl, pH7.4, 1 mM magnesium acetate and protease inhibitors). The homogenates were centrifuged at 800*g* for 10 min at 4 °C and the supernatants were layered on 5 to 50% sucrose gradients prepared in a buffer containing 10 mM Tris–HCl, pH7.4 and 1 mM magnesium acetate. The gradients were centrifuged at 39,000 rpm in a Beckman SW41 rotor for 2.5 h at 4 °C.

### TAP and coimmunoprecipitation and in vitro kinase assay

TAP was carried out as described previously (48). For immunoprecipitation, cells were lysed in CHAPS buffer (40 mM Tris–HCl, pH 7.4, 120 mM NaCl, 1 mM EDTA, and 0.3% CHAPS with complete protease/phosphatase inhibitor cocktail) as previously described (49), with or without digitonin preextraction. The mTORC2 kinase assay was performed as previously described (5), and kinase reactions were carried out at 30 °C for 30 min and then stopped by boiling the mixtures in SDS sample buffer. GST-Akt1 tail (a.a. 280–480) purified from *Escherichia coli* were used as substrates.

## Data availability

All primary data are available upon request.

## Supporting information

This article contains supporting information.

## Conflict of interest

The authors declare no conflict of interest with the contents of this article.
