# Supplementary material for: NOP14-mediated ribosome biogenesis is required for mTORC2 activation and predicts rapamycin sensitivity
Source: J Biol Chem. 2024 Jan 23;300(3):105681. doi: 10.1016/j.jbc.2024.105681 (PMC10891341; doi:10.1016/j.jbc.2024.105681)
Supplement: Supporting Figure S1 [file mmc1.docx]

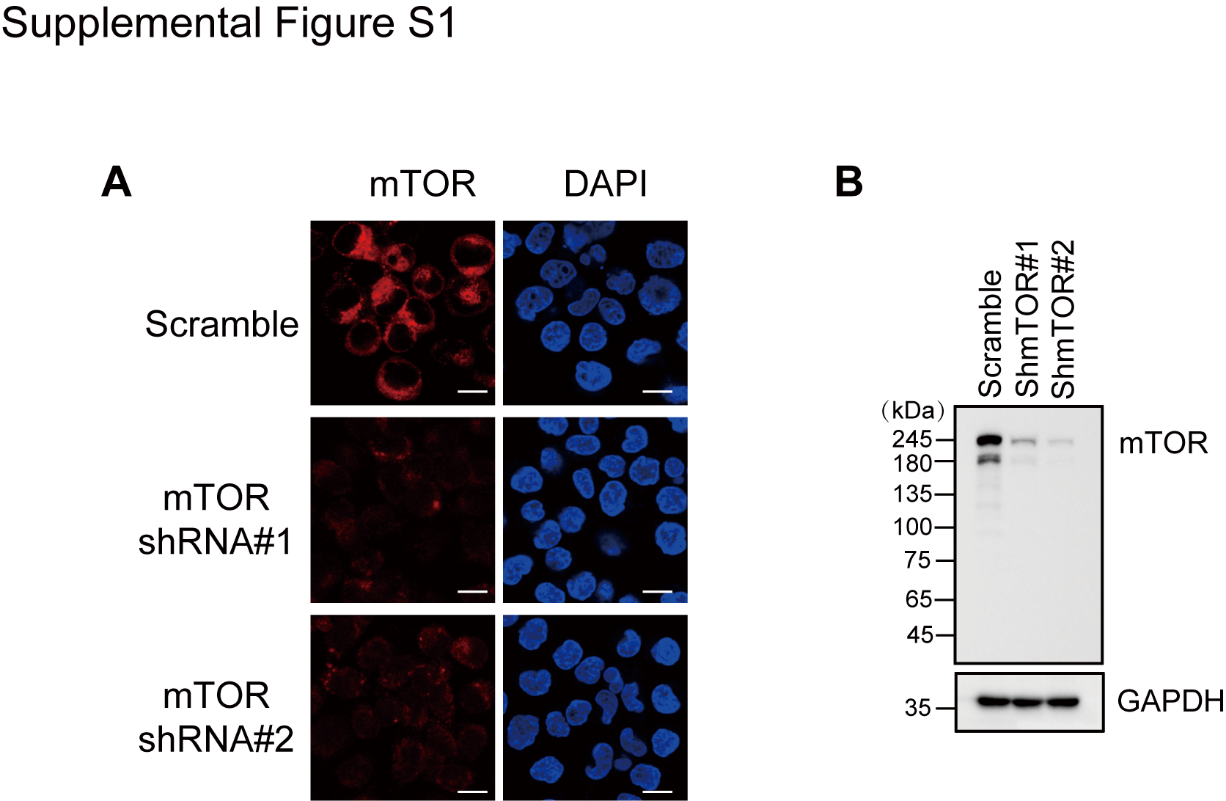


***A,*** Representative immunofluorescence images of mTOR kinase (in red) in HNE1 cells transfected with control shRNA (scramble) or shRNA-targeting mTOR kinase. Scale bar: 20 μm. ***B****,* Western blot analysis of mTOR kinase in HNE1 cells transfected with control (scramble) or mTOR-specific shRNAs. Two shRNAs were used for mTOR knockdown: 1#: 5’- AAGAAGAAGGTAGGGACGCTGAT -3’ and 2#: 5’- GGCCGCATTGTCTCTATCAAGTT -3’.
