# Supplementary material for: NOP14-mediated ribosome biogenesis is required for mTORC2 activation and predicts rapamycin sensitivity
Source: J Biol Chem. 2024 Jan 23;300(3):105681. doi: 10.1016/j.jbc.2024.105681 (PMC10891341; doi:10.1016/j.jbc.2024.105681)
Supplement: Supporting Tables S1 and S2 [file mmc3.docx]

**Supplementary Tables**

**Table S1． Correlation between NOP14 expression and clinicopathologic characteristics of NPC patients**

| **Characteristics** | **NOP14 Expression:**  **No. of Patients (%)**  **Low (82) High (50)** | ***P*-Value^c^** |
| --- | --- | --- |
| **Gender**  Male  Female  **Age, y**  ≦45  >45  **Smoking Status**  No  Yes  **Drinking Status**  No  Yes  **VcA-IgA^a^**  ≦1:320  >1:320  **EA-IgA^a^**  ≦1:80  >1:80  **T Stage^b^**  T1-T2  T3  T4  **N Stage^b^**  N0-N1  N2-N3  **M Stage^b^**  M0  M1  **Clinical Stage^b^**  I-II  III  IV  **Treatment Regimens**  Radiotherapy  Radiochemotherapy  Chemotherapy  Unknown | 64 (61.5) 40 (38.5)  18 (64.3) 10 (35.7)  50 (68.5) 23 (31.5)  32 (54.2) 27 (45.8)  48 (67.6) 23 (32.4)  34 (55.7) 27 (44.3)  66 (65.3) 35 (34.7)  16 (51.6) 15 (48.4)  47 (61.0) 30 (39.0)  34 (65.4) 18 (34.6)  42 (63.6) 24 (36.4)  39 (61.9) 24 (38.1)  25 (69.4) 11 (30.6)  38 (61.3) 24 (38.7)  19 (55.9) 15 (44.1)  54 (71.1) 22 (28.9)  28 (50.0) 28 (50.0)  79 (63.2) 46 (36.8)  3 (42.9) 4 (57.1)  20 (87.0) 3 (13.0)  37 (58.7) 26 (41.3)  25 (54.3) 21 (45.7)  30 (66.7) 15 (33.3)  44 (60.3) 29 (39.7)  3 (42.9) 4 (57.1)  5 (71.4) 2 (28.6) | 0.790  0.093  0.161  0.168  0.616  0.839  0.496  **0.014**  0.497  **0.023**  0.607 |

**a** Data of VcA-IgA and EA-IgA for 3 patients were unknown.

**b** Tumor size, lymph node involvement, distant metastasis and clinical stage were classified according to the seventh edition of the UICC/AJCC staging system.

**c** Two-sided *P*-values were analyzed by Pearson’s Chi-square test or Continuity Correction to evaluate the significance of correlations. Bold print indicates statistical significance.

**Table S2. Multivariate analysis of different prognostic parameters in 132 patients with NPC**

| **Variables** | **DFS**  **HR (95% CI)^c^ *P*^d^** | **DMFS**  **HR (95% CI) ^c^ *P*^d^** | **OS**  **HR (95% CI) ^c^ *P*^d^** |
| --- | --- | --- | --- |
| Gender  Age  Smoking Status  Drinking Status  VcA-IgA**^a^**  EA-IgA**^a^**  T Stage**^b^**  N Stage**^b^**  M Stage**^b^**  Clinical Stage**^b^**  Treatment Regimens  NOP14 expression | 1.15 (0.32-4.13) 0.836  1.54 (0.62-3.80) 0.351  2.37 (0.82-6.89) 0.112  0.58 (0.19-1.83) 0.354  0.90 (0.38-2.14) 0.813  1.27 (0.52-3.14) 0.601  1.28 (0.44-3.71) 0.644  1.29 (0.49-3.42) 0.607  —  **1.89 (1.05**-**3.40) 0.033**  0.58 (0.27-1.24) 0.160  **2.29 (1.04**-**5.04) 0.039** | 2.50 (0.48-13.03) 0.276  1.39 (0.43-4.55) 0.581  2.65 (0.62-11.32) 0.188  0.63 (0.13-3.19) 0.579  0.38 (0.11-1.33) 0.130  1.02 (0.31-3.38) 0.978  1.38 (0.33-5.83) 0.660  **2.20 (1.22**-**3.96) 0.009**  —  1.24 (0.24-6.51) 0.798  0.56 (0.20-1.56) 0.266  2.00 (0.67-6.00) 0.214 | 0.72 (0.23-2.30) 0.579  **2.21 (1.11**-**4.40) 0.024**  1.32 (0.56-3.13) 0.529  0.93 (0.39-2.23) 0.876  0.66 (0.32-1.39) 0.280  1.00 (0.48-2.06) 0.995  0.76 (0.32-1.78) 0.528  0.73 (0.30-1.80) 0.501  1.40 (0.28-7.15) 0.683  **2.01 (1.16**-**3.79) 0.014**  0.71 (0.38-1.33) 0.287  **2.39 (1.22**-**4.67) 0.011** |

**a** Titer of VCA-IgA: ≦ vs. > 1:320; Titer of EA-IgA: ≦ vs. > 1:80

**b** Tumor size, lymph node involvement, distant metastasis and clinical stage were classified according to the seventh edition of the UICC/AJCC staging system.

**c** Adjusted for gender group, age (≦ and > 45 years-old), smoking status at diagnosis, drinking status at diagnosis, titer of VcA-IgA (≦ and > 1:320), titer of EA-IgA (≦ and > 1:80), T stage (T1-T2/T3/T4), N stage (N0-N1/N2-N3), M stage (M0/M1), Clinical stage (I-II/III/IV), treatment regimens (radiotherapy/radiochemotherapy/chemotherapy/unknown), NOP14 expression (low and high expression level).

**d** Cox regression model.
